# Ovarian Stromal Tumor Presenting as Ovarian Torsion: A Case Report

**DOI:** 10.7759/cureus.76640

**Published:** 2024-12-30

**Authors:** Srikaran Bojja, Nismat Javed, Marcos Molina, Harriet Smith, Misbahuddin Khaja

**Affiliations:** 1 Internal Medicine, BronxCare Health System, New York City, USA; 2 Internal Medicine, BronxCare Health System/Icahn School of Medicine at Mount Sinai, New York City, USA; 3 Obstetrics and Gynecology, BronxCare Health System, New York City, USA; 4 Internal Medicine/Pulmonary Critical Care, BronxCare Health System/Icahn School of Medicine at Mount Sinai, New York City, USA

**Keywords:** diagnosis, management, ovarian sex cord-stromal tumor, ovarian torsion, tubo-ovarian mass

## Abstract

Ovarian sex cord-stromal tumors (SCST) are a rare subset of ovarian neoplasms originating from supportive tissues surrounding oocytes. Despite their rarity, prompt diagnosis and management are crucial due to their potential for diverse clinical presentations and the need to optimize patient outcomes. A 25-year-old female patient was initially diagnosed with pyelonephritis but later discovered to have a large right adnexal mass suspected to be a tubo-ovarian abscess. Further evaluation, including tumor markers and imaging, suggested the possibility of a germ cell tumor or sex-cord stromal tumor, leading to surgical intervention. During surgery, a necrotic tubo-ovarian mass with torsion was found and removed, with subsequent pathology confirming a benign stromal tumor. The patient experienced a smooth recovery postoperatively. The management of ovarian SCST, which is rare and has varied clinical presentations, requires accurate diagnosis and a customized treatment approach. These typically benign tumors can produce steroid hormones, leading to distinct symptoms like virilization or estrogen excess. Diagnostic tools include imaging and tumor markers, while surgical options range from conservative to extensive based on specific tumor and patient characteristics. Post-treatment surveillance involves monitoring symptoms and tumor markers. Advancing the understanding and care of these tumors relies on ongoing research and collaborative, multidisciplinary efforts to improve patient outcomes.

## Introduction

Ovarian cancer is the seventh most diagnosed cancer in the world and the eighth most common cause of death in women [1]. Ovarian sex cord-stromal tumors (SCST) encompass a diverse array of both benign and malignant growths originating from the cell population responsible for forming the supportive tissues surrounding oocytes, which are the non-germ cell and non-epithelial elements of the gonads and comprise about 1.2% of all primary ovarian cancers [2]. These tumors represent a relatively rare subset of ovarian neoplasms and are typically classified based on their histological features, including fibromas, thecomas, and fibrothecomas [2].

SCSTs are a diverse group of benign and malignant neoplasms that arise from either ovarian stromal cells or primitive sex cord cells. They are typically classified into three categories: pure stromal tumors, pure sex cord tumors, and mixed SCSTs. Pure stromal tumors originate from mesenchymal cells of the ovarian stroma; examples include fibromas, thecomas, sclerosing stromal tumors, microcystic stromal tumors, Leydig cell tumors, and steroid cell tumors. Pure sex cord tumors develop from primitive sex cord cells and include granulosa cell tumors, Sertoli cell tumors, and sex cord tumors with annular tubules. Mixed SCSTs feature both stromal and sex cord components, encompassing Sertoli-Leydig cell tumors and other SCSTs not otherwise specified [3].

While benign, these tumors can manifest with a variety of clinical presentations, ranging from asymptomatic cases detected incidentally during routine pelvic examinations to symptomatic presentations such as pelvic pain, abdominal distension, or urinary frequency [4]. Despite their generally non-aggressive behavior, prompt diagnosis and appropriate management are essential to mitigate potential complications and optimize patient outcomes. In this report, we present the case of a 25-year-old female patient who presented with left flank pain, hematuria, nausea, and abdominal pain and was subsequently diagnosed with a benign stromal ovarian tumor. Through this case, we aim to discuss the diagnostic evaluation, treatment approach, and clinical course of benign stromal ovarian tumors, highlighting the importance of a multidisciplinary approach in managing these rare but clinically significant entities. 

## Case presentation

A 25-year-old female patient, with a past medical history of nephrolithiasis that had resolved, presented to the hospital with left flank pain, hematuria, nausea, and abdominal pain. The patient was evaluated in the emergency department, including labs notable for slight leukocytosis (white blood cell count: 11.8; reference range: 4800-10800/uL) and lactic acidosis (2.0 mmoles/L; reference range: 0.5-1.6 mmoles/L). Urinalysis showed positive nitrates, moderate bacteria, moderate number of white blood cells, and red blood cells, likely due to the patient being on her period at admission. Urine culture showed 50,000-100,000 CFU/mL of Group B *Streptococcus*. The patient was admitted to the medicine service for treatment of pyelonephritis and pain control. 

**Figure 1:**
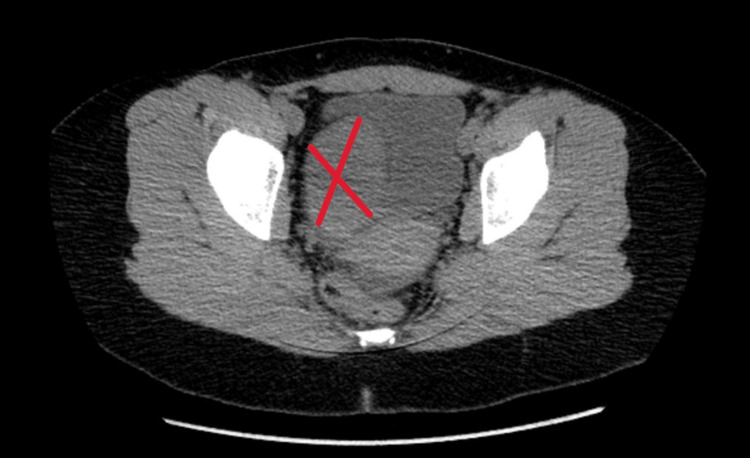
CT scan of the abdomen and pelvis showing a 6.6 x 9.2 x 10.0 cm adnexal mass (red cross). This mass is distinguished from the surrounding tissue by its size, shape, and the absence of typical ovarian follicular structure, suggesting the presence of a complex solid-cystic lesion

The CT abdomen/pelvis with/without contrast revealed an approximately 6.6 x 9.2 x 10.0 cm right adnexal mass (Figure 1).

**Figure 2:**
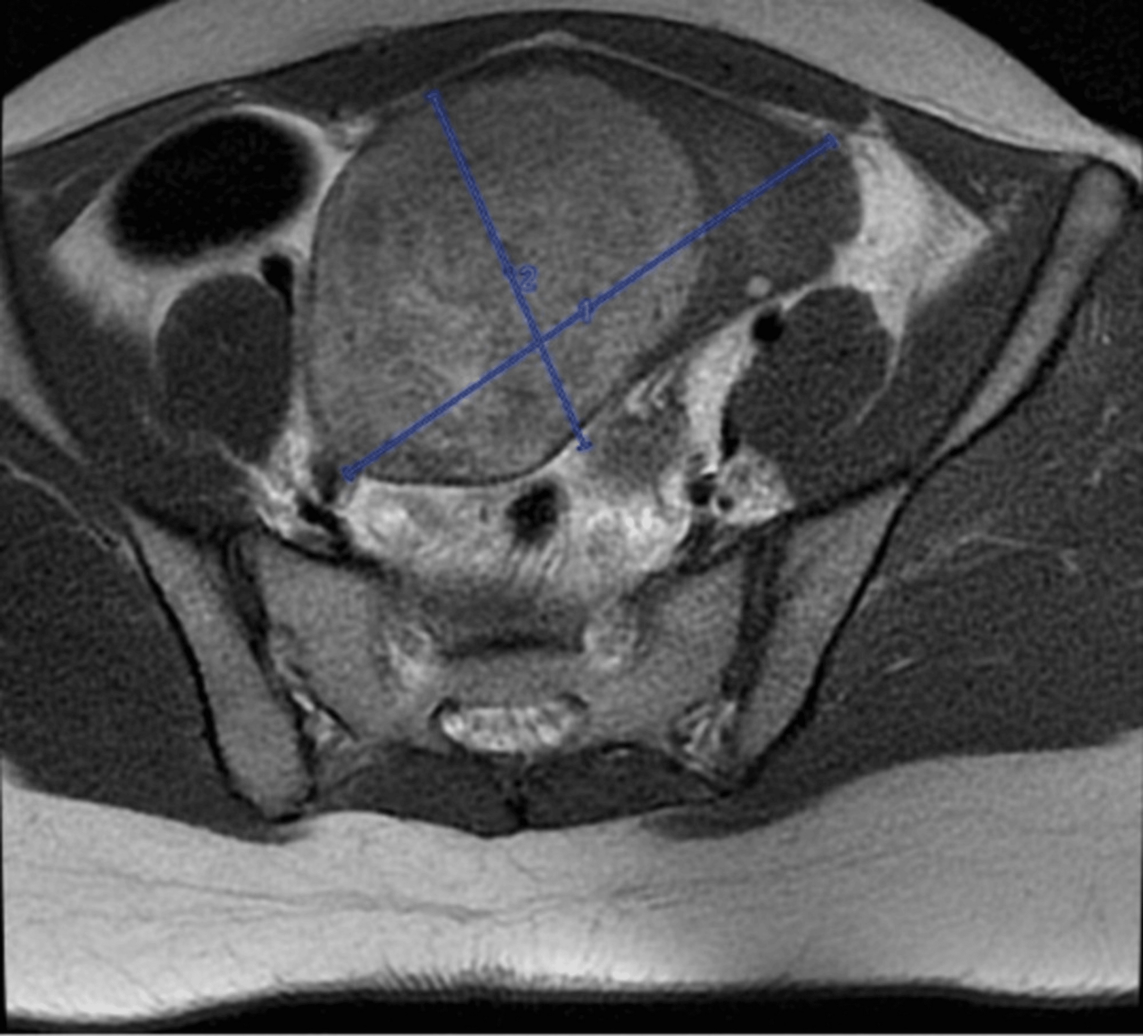
Pelvic MRI (coronal view) highlighting the extent of the solid mass across the right adnexal region into the left adnexal region, indicating its large size and complex nature with tiny cystic components

**Figure 3:**
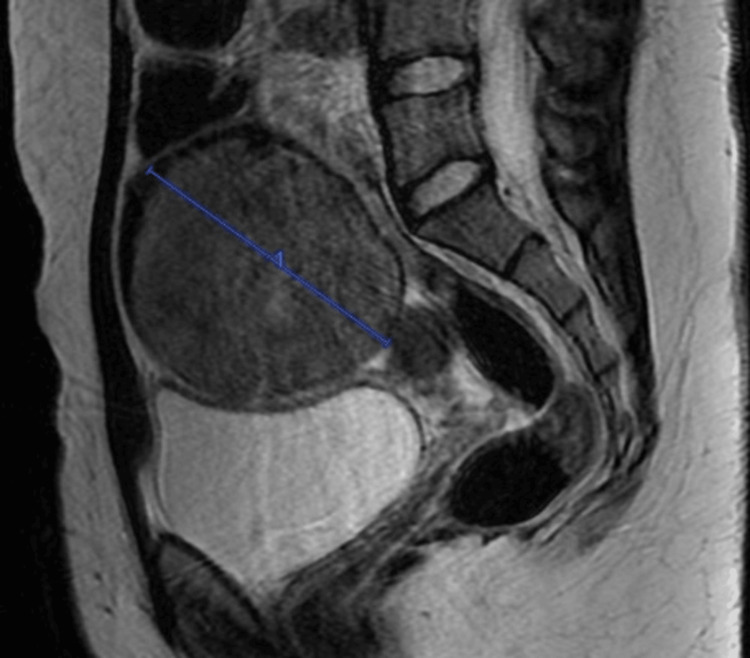
MRI (sagittal view) showing a 7.6 x 8.7 x 11.6 cm solid mass with tiny cystic components in the region of the right adnexa that crosses the midline into the left adnexal region

The Medicine team consulted the Obstetrics and Gynaecology service for the mass and recommended adding doxycycline/metronidazole to IV ceftriaxone to treat for suspected tubo-ovarian abscess. Additionally, recommendations included the collection of a sexually transmitted infections (STI) panel, tumor markers, pelvic ultrasound, and pelvic MRI. An STI panel came back negative. The MRI showed a large 7.6 x 8.7 x 11.6 cm complex predominantly solid mass with tiny cystic components in the region of the right adnexa that crosses the midline into the left adnexal region (Figures 2, 3).

**Figure 4:**
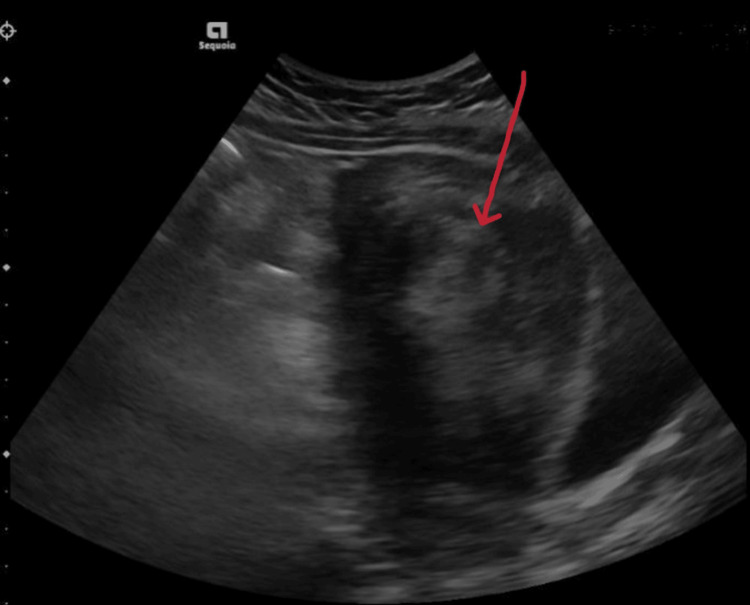
Transvaginal ultrasound showing solid appearing lesion in right adnexa (red arrow)

A transvaginal pelvic ultrasound was done that showed an 8.3 x 1.2 x 7.5 cm solid-appearing lesion visualized within the right adnexa (Figure 4). 

Tumor markers were notable for elevated lactate dehydrogenase (355) and elevated testosterone (121); all others, including human chorionic gonadotropin (HCG), alpha-fetoprotein, and carcinoembryonic antigen, were unremarkable. Based on the tumor markers (lactate dehydrogenase 355), MRI results, and clinical presentation (pain, possible rapid growth, and iron-deficiency anemia), the mass was estimated to be consistent with germ cell tumor (GCT) or SCST. 

**Figure 5:**
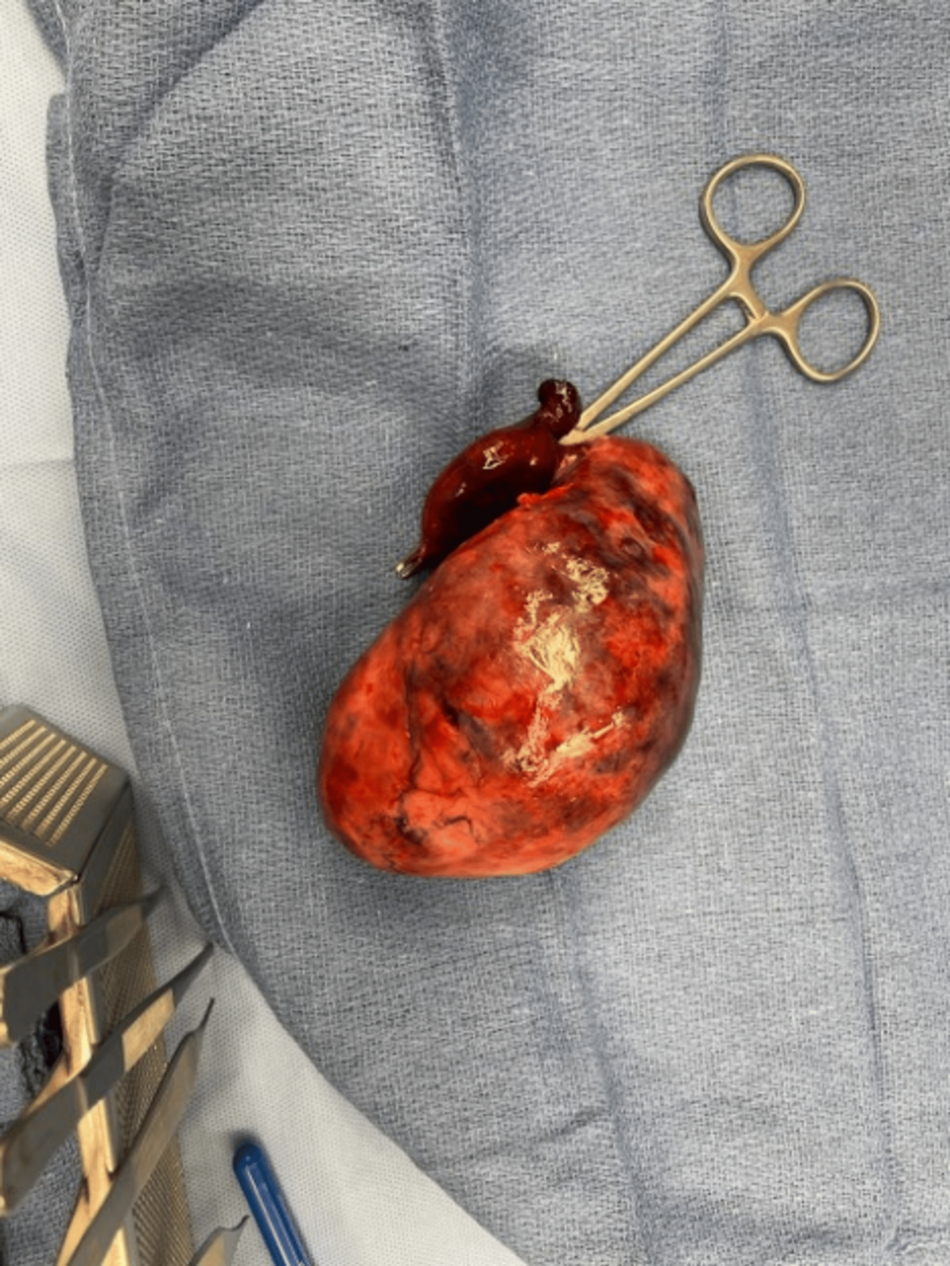
Swollen and hemorrhagic ovary visualized with fallopian tube intraoperatively

The surgical plan initially involved a laparoscopic approach to remove an abdominal mass. Upon visualization, the mass appeared swollen and hemorrhagic, with the colon adhered to it. After separating the colon, the mass remained immobile, obstructing the view of the pedicle as it was fixed over the uterus and round ligaments (Figure 5).

Due to these findings and abnormal tumor markers, the procedure was converted to a laparotomy. During the open surgery, a left tubo-ovarian mass was identified, with a pedicle twisted three times, resulting in a necrotic fallopian tube. The mass was removed and sent for a frozen section, which showed extensive tissue necrosis and stromal cells without malignancy. To prevent future complications, a right oophoropexy was performed. The patient recovered well postoperatively, with pain improvement, and was discharged after a few days. Final pathology confirmed the tumor as a benign stromal tumor, with no malignant cells detected. 

## Discussion

Ovarian SCSTs originate from dividing cells, typically forming specialized gonadal stroma surrounding oocytes, such as granulosa cells, theca cells, Sertoli cells, Leydig cells, and fibroblasts. In contrast, the two other major types of ovarian neoplasms originate from germ cells or dividing cells that give rise to Müllerian epithelium, ovarian surface epithelium, or fallopian tube epithelium [2]. These tumors are generally non-cancerous and encompass various subtypes, including but not limited to ovarian fibromas, thecoma, and fibrothecomas, each characterized by distinct histological features and clinical presentations [2]. Understanding the classification of these tumors is crucial for accurate diagnosis and appropriate management. These tumors encompass a spectrum of histological subtypes, including fibromas, thecomas, and fibrothecomas, each characterized by distinct cellular compositions and architectural patterns [2]. Unlike malignant ovarian tumors, benign stromal tumors typically lack aggressive features such as invasion into surrounding tissues or metastasis to distant organs. However, they can present with symptoms such as pelvic pain, abdominal distension, or urinary frequency, necessitating accurate diagnosis and appropriate management strategies [4]. 

Malignant ovarian SCSTs make up less than 8% of malignant ovarian neoplasms [5]. The average age at diagnosis is 50 years; 12% of patients were <30 years and 57% were between 30 and 59 years [5]. Morphologically, SCSTs typically exhibit distinct gonadal stromal features such as granulosa, theca, Sertoli, or Leydig cell characteristics. Additionally, they may manifest with less specific morphological traits like fibroblastic features. They can encompass regions displaying other types of stromal differentiation, such as cartilage or skeletal muscle, or even areas of epithelial differentiation known as "heterologous elements" [5]. SCSTs are rarer than epithelial cells and germ cell origin, representing only about 1.2% of all ovarian cancers [6].

The FIGO (International Federation of Gynecology and Obstetrics) staging system for ovarian cancer categorizes the disease based on the extent of spread within the body [1]. In stage I, the cancer is confined to the ovaries, with subcategories reflecting the exact location and involvement. Stage II denotes the cancer's spread beyond the ovaries but still within the pelvis, potentially affecting nearby tissues such as the fallopian tubes or uterus. Stage III signifies the cancer's progression to the abdominal cavity or nearby lymph nodes, indicating a more advanced disease. Finally, in stage IV, ovarian cancer has metastasized to distant organs such as the liver or lungs, indicating the most severe stage with widespread dissemination. The FIGO staging system helps guide treatment decisions and prognosis assessment for individuals diagnosed with ovarian cancer, aiding in the development of personalized and effective management strategies. [1] Survival is 80-90% in early stages (FIGO stage I or II) compared to 25% in later stages (FIGO stage III or IV) [3]. 

Patients with ovarian SCSTs typically present with nonspecific abdominal or pelvic symptoms or as an incidental finding [2,5]. Some SCSTs produce steroid hormones, leading to specific signs such as virilization or estrogen excess [2,5]. Symptoms of estrogen excess include abnormal uterine bleeding and endometrial neoplasms, while signs of androgen excess may include hirsutism, acne, and menstrual irregularities [2,5]. Additionally, ovarian fibromas or luteinized thecoma with sclerosing peritonitis can present with ascites, which might cause bowel obstruction [2,5]. Because the symptoms are so variable, ovarian cancer should be considered in premenopausal women with unexplained ovarian enlargement or postmenopausal women with a palpable ovary or mass [7]. Some patients with specific SCST types, such as ovarian fibroma, may present with ascites and/or pleural effusion, potentially associated with Meigs syndrome [5]. One study found that serum levels of cancer antigen 125 (CA125), human epididymis protein 4 (HE4), and carcinoembryonic antigen (CEA) did not significantly differ between benign and malignant tumors, indicating these markers were not effective for preoperative differentiation [5,8]. Ovarian fibromas appear as solid hypoechoic masses on ultrasound, showing diffuse, slightly hypoattenuating characteristics on CT scans, and exhibit low signal intensity on both T1- and T2-weighted MR images, with occasional high-signal-intensity areas indicating edema or cystic degeneration [9]. Sclerosing stromal tumors, rare in young women, show varied signal intensities on MRI and marked early enhancement, which helps distinguish them from other neoplasms [10]. There is no consensus on the size ranges of a large vs huge ovarian tumor, but most tumors between 5 and 15 cm are termed large, and tumors over 20 cm are termed huge/giant [7,11]. The most useful biomarker for identifying SCST is α-inhibin, which is positive in most neoplasms in the sex cord-stromal group [12]. 

We conducted a literature review of cases that presented with ovarian torsion as a result of similar tumors [13-16].

**Table 1:** Summary of cases reviewed

Author	Age/sex	Symptoms	Type of Tumor	Findings	Management	Outcome
Tligui et al. [13]	39 years/female	Abdominal Pain	Sertoli-Leydig	Ultrasound showed torsion	Salpingo-oophorectomy	Alive
Maleki et al. [14]	35 years/pregnant female	Nausea and vomiting	Thecoma	Ultrasound did not visualize right ovary	Tumor detorsion and resection	Alive
Ahmed et al. [15]	46 years/female	Abdominal Pain	Teratoma	Ultrasound showed adnexal mass	Antibiotics as patient declined surgery	Alive
Kojima et al. [16]	32 years/female	Abdominal Pain	Recurrence of Microcystic tumor	Ultrasound showed multiple masses	Surgery	Alive

The patient in the current case was younger as compared to the patients presenting with ovarian torsion as a result of these tumors previously. Additionally, it was also noted that pregnancy is a risk factor for ovarian torsion [14]. The majority of the cases presented similar vague symptoms of abdominal pain and not hypersecretion from the tumor. While management strategies were primarily surgical, one case did not have the same strategy due to patient preference [15]. Interestingly, in another case, the patient had a history of prior fertility-preserving surgical treatment but developed a recurrence that led to mutation analysis and further diagnosis of a syndrome around the somatic mutation CTNNB1 [16]. 

Management strategies for SCSTs of the ovary include a spectrum of approaches tailored to the individual's condition and preferences, including surgical interventions, adjuvant chemotherapy, hormonal therapy, and other systemic agents like antiangiogenic therapy [17]. 

Surgical options can range from conservative surgeries aimed at preserving fertility to more aggressive interventions for advanced malignancies [17]. Adjuvant therapies may involve chemotherapy, with various regimens available based on the patient's specific situation. Hormonal therapy and other systemic treatments, such as antiangiogenic agents, offer additional avenues for targeting these tumors. Radiation therapy is considered on a case-by-case basis. Due to the unique characteristics of each case and the rarity of these tumors, post-treatment surveillance is customized, focusing on monitoring symptoms, physical examinations, and tumor marker levels when applicable [17]. 

Surgical interventions for SCSTs of the ovary are primarily dependent on the tumor's nature and patient preferences. For benign tumors, procedures like oophorectomy or ovarian cystectomy are preferred to preserve fertility, especially in younger patients. However, in postmenopausal women, especially those with thecomas, a total hysterectomy with bilateral salpingo-oophorectomy might be advised due to potential estrogenic effects and the risk of endometrial neoplasms. In cases of malignant SCSTs, the surgical approach is more comprehensive, involving surgical staging and potentially more extensive procedures. The choice of surgery, whether conservative like unilateral oophorectomy or more radical like total hysterectomy, hinges on multiple factors, including the patient's age, fertility desires, and the tumor's specifics. These decisions are often made intraoperatively, based on real-time histologic evaluations, and aligned with the patient's treatment goals and quality of life considerations [17]. 

## Conclusions

The atypical age of presentation, initially misleading clinical symptoms resembling pyelonephritis, and the complex nature of the tumor with necrosis and torsion leading to fallopian tube involvement highlights the diagnostic challenges and surgical complexities inherent in such cases. In our case, the patient was younger, which led to a focus on fertility-preserving surgical treatment. Despite these challenges, the multidisciplinary approach involving medical, surgical, and diagnostic teams facilitated prompt diagnosis and appropriate management, ultimately resulting in a favorable outcome for the patient. This case underscores the importance of thorough evaluation, consideration of differential diagnoses, and individualized treatment strategies tailored to the patient's unique circumstances.

Continuing research and collaboration among healthcare professionals will enhance our understanding and management of rare ovarian stromal tumors, ultimately improving patient outcomes to prevent mortality. Routine, standardized assessment of patient-reported symptoms for a pelvic mass can lead to earlier detection of subtle changes, more individualized treatment planning, stronger communication and shared decision-making, ongoing monitoring of treatment effectiveness, standardized data collection, and better identification of psychosocial needs. For example, earlier reports of persistent pelvic pain despite treatment of acute causes should raise suspicion on the part of the primary care provider to coordinate with earlier gynecology specialists, and in the meantime, investigations should include transvaginal ultrasound with serum markers to rule out further diagnoses. Additionally, this diagnosis has to be highlighted in patients with prior surgical history as seen in some case reports. 
